# A Prospective Observational Study Comparing Hybrid Acrylic and Collamer Posterior Chamber Phakic Intraocular Lenses for High Myopia

**DOI:** 10.7759/cureus.112632

**Published:** 2026-07-14

**Authors:** Gaurav Pande, Virendra P Singh, Priyanka, Rajendra P Maurya, Asha Devi, Sanjay K Bosak, Abdullah Al-Mujaini

**Affiliations:** 1 Department of Ophthalmology, Narayana Nethralaya, Bengaluru, IND; 2 Department of Ophthalmology, Banaras Hindu University Institute of Medical Sciences, Varanasi, IND; 3 Department of Ophthalmology, Sultan Qaboos University College of Medicine &amp; Health Sciences, Seeb, OMN

**Keywords:** high myopia, implantable collamer lens, implantable phakic contact lens, refractive surgery, visual outcomes

## Abstract

Purpose: To evaluate clinical outcomes following implantation of two types of posterior chamber phakic intraocular lenses: a reinforced hydrophilic acrylic implantable phakic contact lens IPCL (model V2.0; Care Group, Baroda, India) and a Collamer implantable phakic contact lens Visian ICL (model V4c; Staar Surgical Co., Monrovia, CA) for the treatment of high myopia (more myopic than ˗5.00 Dioptre) in adults.

Objective: To compare visual and refractive outcomes and postoperative complications between a reinforced hydrophilic acrylic implantable phakic contact lens IPCL and a Collamer implantable phakic contact lens Visian ICL for the treatment of high myopia and myopic astigmatism in adults.

Methodology: This is a prospective, observational study in a tertiary ophthalmic care centre in Varanasi, India. Fifty-five eyes from 55 patients with high myopia or myopic astigmatism were included in the study. Group A (27 eyes) received implantable phakic contact lens IPCLs, and Group B (28 eyes) received implantable Collamer lens ICLs. All patients underwent preoperative assessments, including optical biometry, keratometry, axial length, central corneal thickness, and white-to-white measurement. Postoperative evaluations were conducted at 1, 6, and 12 months, assessing uncorrected visual acuity (UCVA) and best-corrected visual acuity (BCVA), refractive stability, and adverse effects.

Results: Both groups showed statistically significant improvement in visual acuity over 12 months. Preoperative mean LogMAR BCVA was 0.411 ± 0.191 (Group A) and 0.366 ± 0.277 (Group B). At 12 months, mean postoperative LogMAR UCVA was 0.268 ± 0.202 (Group A) and 0.146 ± 0.133 (Group B). Anterior uveitis with intraocular pressure elevation occurred in four eyes (14.8%) in Group A and four eyes (14.3%) in Group B at one week. Glare and halos were reported in eight eyes (29.6%) in Group A and six eyes (21.4%) in Group B during late follow-up.

Conclusion: Both the IPCL and ICL are effective and safe options for correcting high myopia. ICL showed superior visual outcomes as compared to IPCL with comparable complication rates. Though affordability was not assessed, the reinforced acrylic lens remained a potentially lower-cost material, offering meaningful refractive correction.

## Introduction

The incidence of myopia is steadily increasing worldwide, with particularly notable trends observed in Asia. In India, population-based studies have revealed a rise in myopia prevalence 4.6% in rural areas in 1980 to 6.8% in the 2009-2019 period. In urban populations, the prevalence increased from 7.9% to 8.9% over the same timeframe [1]. This trend reflects a significant increase in visual disability related to uncorrected refractive error, which carries substantial social, financial, and developmental consequences.

In cases of high myopia where corneal refractive procedures are contraindicated due to factors such as inadequate corneal thickness or irregular topography, phakic intraocular lenses (P-IOLs) offer a viable surgical alternative. Unlike corneal ablative techniques - such as photorefractive keratectomy (PRK), laser-assisted in situ keratomileusis (LASIK), or small incision lenticule extraction (SMILE) - P-IOL implantation preserves the structural integrity of the cornea by avoiding stromal ablation and utilizing a minimally invasive clear corneal incision [2,3].

P-IOLs are designed to be placed in various intraocular positions: they may be angle-supported, iris-fixated, or situated in the posterior chamber at the ciliary sulcus. A key advantage of this approach is the preservation of the natural crystalline lens, maintaining accommodation and minimizing the risks associated with lens extraction, such as vitreoretinal complications [2].

Earlier generations of P-IOLs, including angle-fixated and iris-supported models, were eventually phased out due to complications such as corneal endothelial loss, uveitis-glaucoma-hyphema (UGH) syndrome, and progressive iris atrophy [2]. These limitations prompted the development of posterior chamber P-IOLs, which offer a greater distance from the corneal endothelium and are thus associated with a reduced risk of endothelial damage [4]. Additionally, design enhancements such as central and peripheral holes have improved aqueous humour dynamics, reducing the risk of postoperative intraocular pressure elevation.

Despite these advancements, comparative clinical data between current P-IOL designs remain limited. To the best of our knowledge, few studies have evaluated visual and safety outcomes between newer posterior chamber P-IOLs. The present study was designed to address this gap. We conducted a prospective, observational study comparing two such lenses in the surgical correction of high myopia.

In this study, we aimed to compare the clinical performance of two contemporary posterior chamber P-IOLs used for the surgical correction of high myopia. Group A received a reinforced hybrid hydrophilic acrylic lens with six haptic pads, a central hole measuring 380 µm, and four paracentral holes. Group B received a Collamer-based lens with four haptic pads, a 360 µm central hole, and two paracentral holes. The primary objective was to evaluate and compare the two lenses in terms of uncorrected visual acuity (UCVA) and best-corrected visual acuity (BCVA), refractive stability, and incidence of postoperative complications over a 12-month follow-up period.

## Materials and methods

This prospective, observational study was conducted at the Regional Institute of Ophthalmology, Institute of Medical Sciences, Banaras Hindu University, Varanasi, India, over 20 months (October 2022 to May 2024). The study adhered to the Declaration of Helsinki and was approved by the ethical committee of Banaras Hindu University (Approval No. Dean/2022/EC/3864, dated 15/04/2023). Written informed consent was obtained from all participants following an explanation of the procedure.

A total of 55 eyes from 55 patients with high myopia of spherical equivalent greater than ˗5.00 Dioptre were included. Group A (27 eyes) received implantable phakic contact lens IPCLs (model V2.0; Care Group, Baroda, India), and Group B (28 eyes) received implantable Collamer lens ICLs (model V4c; Staar Surgical Co., Monrovia, CA). Groups were matched for baseline ocular parameters. Of the total 55 patients, 23 were male (9 in Group A, 14 in Group B), and 32 were female (18 in Group A and 14 in Group B).

Inclusion criteria included age over 21 years, high myopia, more myopic than spherical equivalent ˗5.00 D, stable refraction (<0.50 D change over one year), and unsuitability for corneal refractive surgery. Exclusion criteria were prior intraocular surgery, myopic degeneration, corneal or macular pathology, cataract, glaucoma, uveitis, trauma, shallow anterior chamber, or active ocular infections.

All patients underwent a comprehensive ophthalmic workup. Objective refraction was performed with an autorefractometer (Topcon KR-800, Japan), and refined subjectively for BCVA using a Snellen chart. Slit-lamp examination was followed by fundus biomicroscopy with a 78D lens and peripheral retinal evaluation using indirect ophthalmoscopy and scleral indentation with a 20D lens. Optical biometry (Carl Zeiss Meditech AG, IOL Master 700, Germany) was used to measure keratometry, axial length (AL), and anterior chamber depth (ACD). Central corneal thickness (CCT) was assessed using Cirrus HD-OCT (Carl Zeiss Meditech, Germany) with pachymetry, and white-to-white (WTW) diameter was measured using digital callipers at the slit lamp. Intraocular pressure was recorded by Goldmann applanation tonometry.

P-IOL power was calculated using the manufacturer's modified vertex formula to target emmetropia. Lens size selection was based on ACD and WTW values. The same surgeon performed all measurements for consistency. Eyes with ≤ ˗1.00 D of cylindrical error received non-toric lenses (15 in Group A, 12 in Group B); the remainder received toric implants.

Surgical technique

All procedures were performed by a single experienced surgeon with over 25 years of specialization in refractive surgery. Surgical technique was consistent for both P-IOL types. To minimize intraoperative cyclotorsion, horizontal axis markings were placed at the slit lamp with the patient upright. Pupillary dilation was achieved using 0.8% tropicamide and 5% phenylephrine, 30 minutes prior to surgery.

After aseptic preparation with 5% povidone-iodine, surgery was performed under topical anesthesia (2% lignocaine jelly). Two side-port incisions were created using a 20-G angled microvitreoretinal (MVR) blade. Intracameral plain lidocaine (oculon 1%) and a dispersive ophthalmic viscosurgical device (2% hypromellose) were used to maintain anterior chamber stability. A 3.0 mm temporal clear corneal incision was made at the steepest meridian, and the P-IOL was injected into the anterior chamber, unfolded, and positioned with footplates tucked beneath the iris plane.

Viscoelastic was removed using bimanual irrigation and aspiration, and 2% pilocarpine was instilled to constrict the pupil. Wound integrity was verified via stromal hydration. For toric lenses, intraoperative axis alignment was performed using a Mendez ring to ensure accurate orientation to the pre-marked references. Second-eye surgery was scheduled one to two weeks after the first operation.

Postoperative management

Patients received 1% prednisolone acetate eye drops tapered over a period of four to six weeks, along with 0.5% moxifloxacin eye drops for infection prophylaxis. Cycloplegics were maintained with 1% cyclopentolate hydrochloride for one week postoperatively. Visual acuity and refractive outcomes were evaluated at 1, 6, and 12 months postoperatively. Adverse events were also recorded.

Statistical analysis

Data analysis was conducted using the Statistical Product and Service Solutions (SPSS, version 25.0; IBM SPSS Statistics for Windows, Armonk, NY). Categorical variables were presented in numbers and percentages, and continuous variables were expressed in Mean±SD.

Intergroup comparison was done by the Mann-Whitney U test, while intragroup comparison was done using the Wilcoxon signed-rank test. Correlation between two variables was done using linear regression analysis. The test was referenced for a two-tailed p-value. A p-value <0.05 was considered statistically significant.

## Results

A total of 55 eyes from 55 patients with high myopia, more than -5.00D, were included in the study. Group A (27 eyes) received a reinforced hybrid acrylic P-IOL (IPCL), and Group B (28 eyes) received a Collamer P-IOL (Visian ICL). The mean age in Group A was 23.70 ± 2.76 years and 24.78 ± 3.61 years (p = 0.5823) in Group B, showing no significant difference. Of the total 55 patients, 23 were male (9 in Group A, 14 in Group B), and 32 were female (18 in Group A and 14 in Group B).

Both groups demonstrated significant improvement in UCVA following surgery at 1, 6, and 12 months, as shown in Tables 1-2. In Group A, the mean preoperative LogMAR-UCVA was 1.411 ± 0.121, and preoperative LogMAR-BCVA was 0.411 ± 0.191. In Group B, the mean preoperative LogMAR-UCVA was 1.366 ± 0.217, and preoperative LogMAR-BCVA was 0.366 ± 0.277. Mean post-operative spherical equivalent was -0.20D to +0.20D in IPCL Group A and -0.10D to +0.10D in ICL Group B. Refractive predictability of eyes within ±0.50D of target was 70-90% in IPCL Group A and 80-95% in ICL Group B. Refractive predictability of eyes within ±1.00D of target was 90-100% in IPCL Group A and 95-100% in ICL Group B. At 12 months postoperatively, the Mean LogMAR-UCVA improved to 0.268 ± 0.202 in Group A and 0.146 ± 0.133 in Group B. Within-group comparison showed statistically significant improvement in UCVA from baseline in both groups (p < 0.001, Wilcoxon signed-rank test). Intergroup comparison at 12 months revealed significantly better UCVA in Group B compared to Group A (p = 0.0477, Mann-Whitney U test).

**Table 1:** Preoperative and postoperative visual acuity outcomes in Group A UCVA: uncorrected visual acuity

Visual Acuity	Group A (Mean ± SD)	p value	t-value	F-value
Preop UCVA at baseline	1.411 ± 0.121	Baseline	-	-
Post op UCVA at 1 month	0.308 ± 0.203	0.00164	3.51	12.34
Post op UCVA at 6 months	0.280 ± 0.203	0.00008	4.67	21.82
Post op UCVA at 12 months	0.268 ± 0.202	<0.00001	>5.46	> 29.82

**Table 2:** Preoperative and postoperative visual acuity outcomes in Group B UCVA: uncorrected visual acuity

Visual Acuity	Group B (Mean ± SD)	p value	t-value	F-value
Preop UCVA at baseline	1.366 ± 0.217	Baseline	-	-
Post op UCVA at 1 month	0.311 ± 0.208	0.01878	2.50	6.25
Post op UCVA at 6 months	0.231 ± 0.182	0.0001	4.56	20.77
Post op UCVA at 12 months	0.146 ± 0.133	<0.00001	>5.41	> 29.32

**Figure 1:**
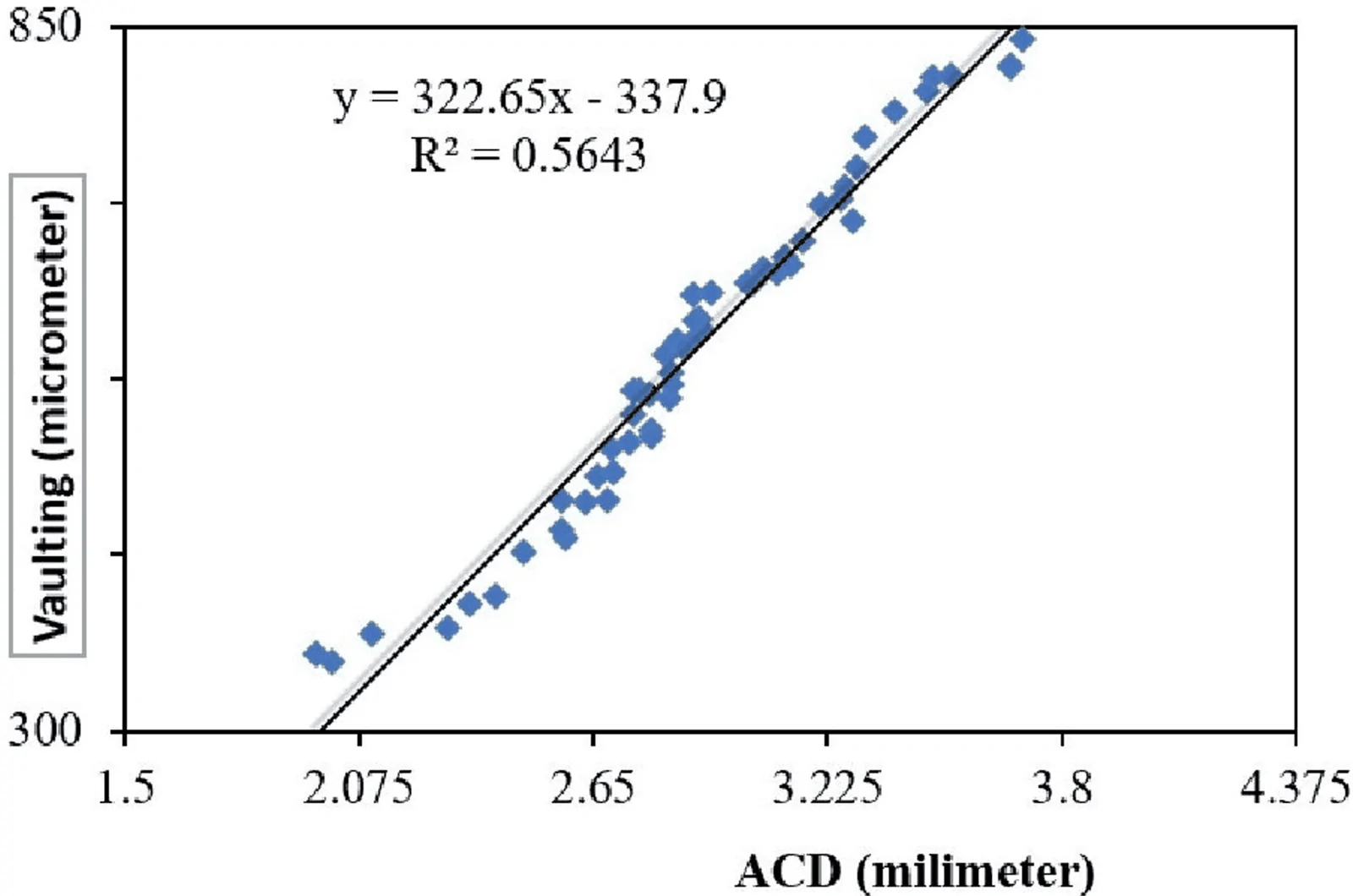
Correlation of anterior chamber depth (mm) with postoperative vaulting (μm) ACD = anterior chamber depth

The mean ACD from corneal endothelium to the anterior lens capsule, measured preoperatively using optical biometry (IOL master 700; Zeiss Group, Oberkochen, Germany), was 2.903 ± 0.390 mm across both groups. The mean postoperative vault, assessed one month after surgery via anterior segment OCT, was 598.89 ± 129.38 µm. Linear regression analysis showed a strong positive correlation between ACD and postoperative vaulting (R2 = 0.5643; p < 0.001), indicating that greater preoperative ACD was associated with higher vaults (Figure 1).

**Figure 2:**
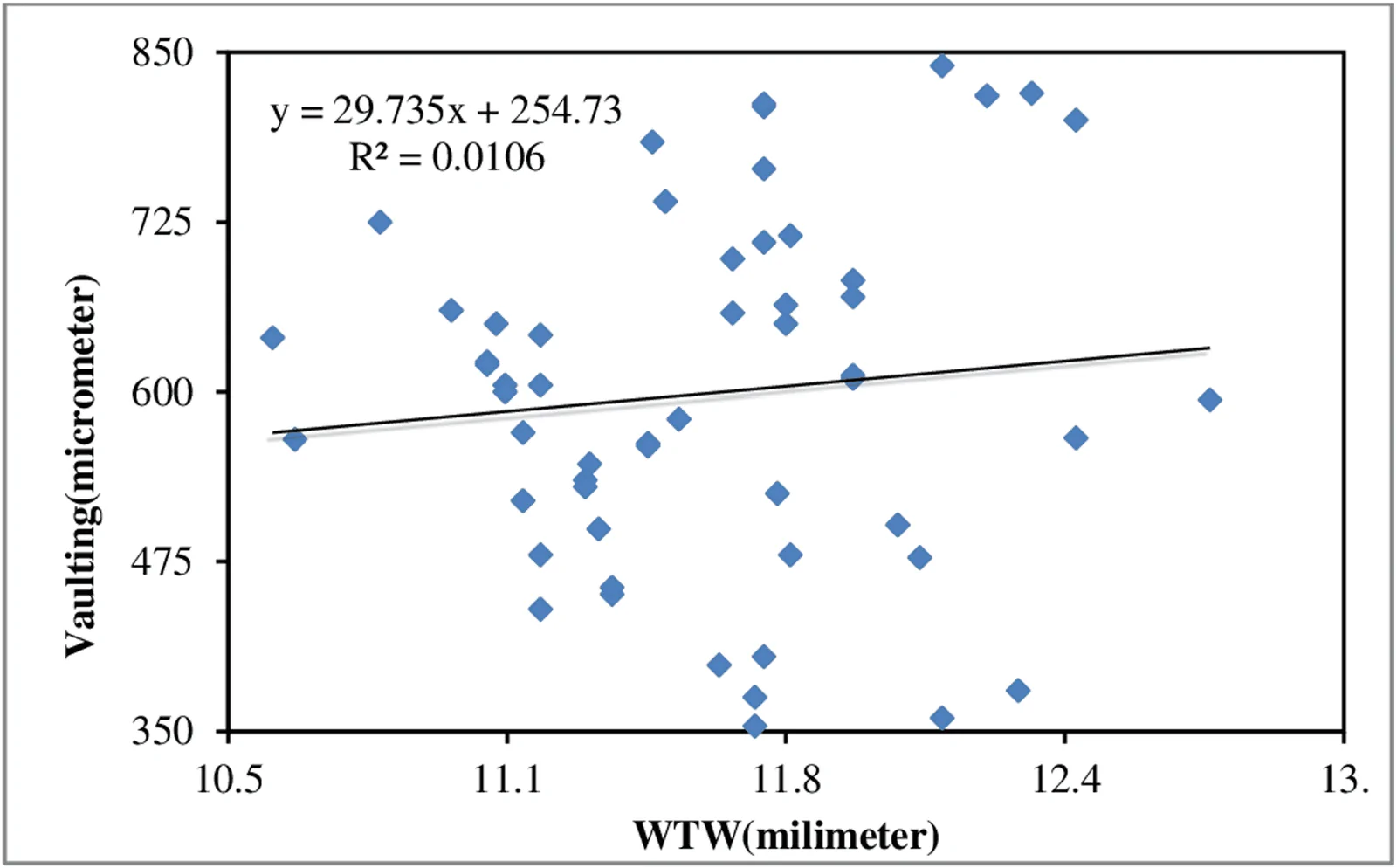
Correlation of white-to-white distance (mm) with postoperative vaulting (μm) WTW = white-to-white measurement

In contrast, WTW corneal diameter, with a mean of 11.574 ± 0.447 mm, was not significantly correlated with vault height (R2 = 0.0106; p = 0.4548) (Figure 2).

No cases of sight-threatening complications, such as corneal edema, retinal detachment, endophthalmitis, pupillary block glaucoma, lens opacity, or chronic inflammation, were observed during the 12-month follow-up in either group.

Eight eyes (14.5%) developed acute anterior uveitis and transient intraocular pressure elevation within one week postoperatively -four eyes (14.8%) in Group A and four eyes (14.3%) in Group B. These were successfully managed with topical corticosteroids (prednisolone acetate 1%), cycloplegics (cyclopentolate hydrochloride 1%), and intraocular pressure-lowering agents (timolol maleate 0.5%) over three weeks.

**Figure 3:**
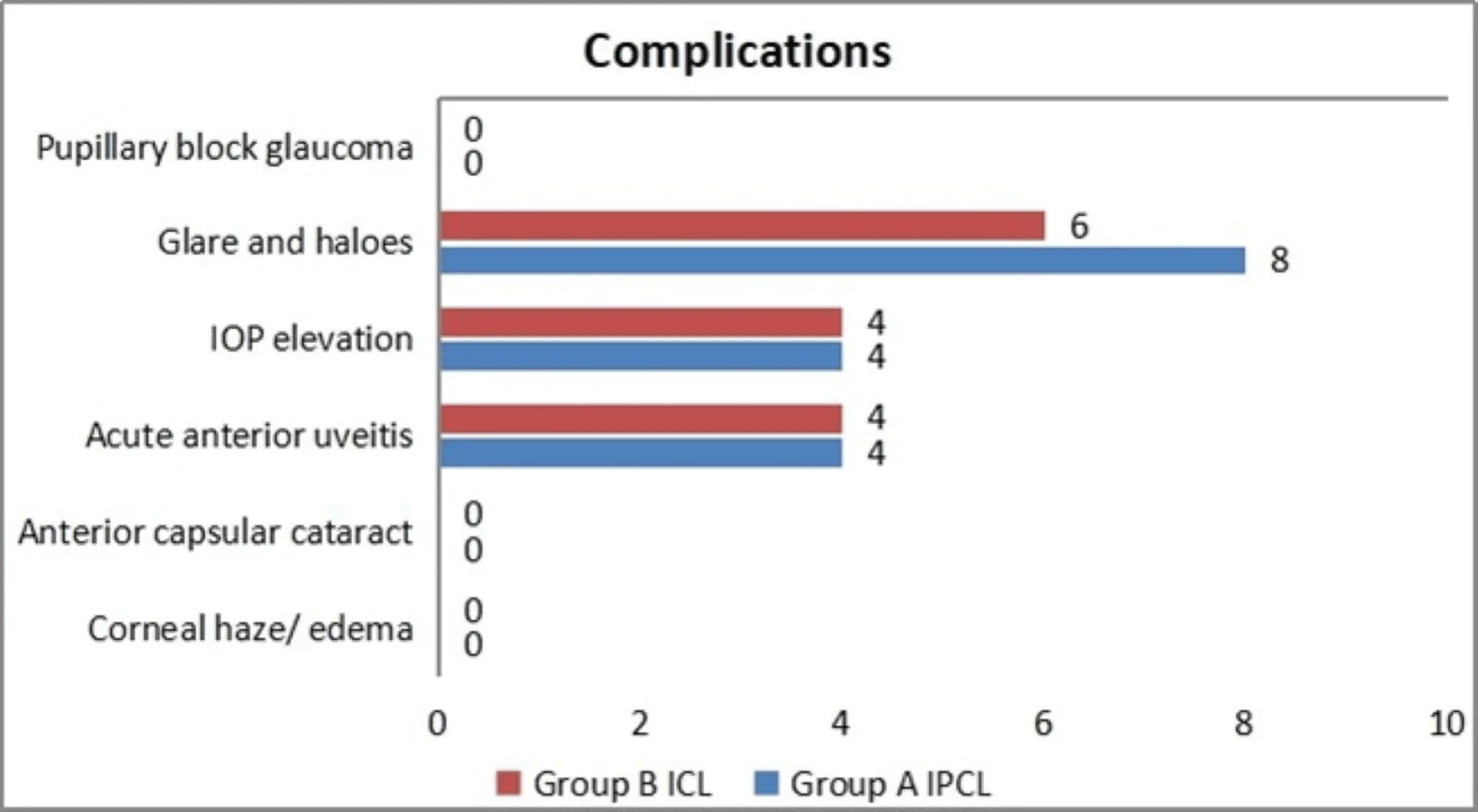
Complications reported by patients from both groups in the late postoperative period

Fourteen eyes (25.4%) reported glare and halos during the late postoperative period: eight eyes (29.6%) in Group A and six eyes (21.42%) in Group B (Figure 3).

## Discussion

P-IOLs offer a valuable alternative to corneal refractive procedures, particularly in patients with high myopia or contraindications to keratorefractive surgery. These lenses correct refractive errors without compromising accommodation, as they preserve the crystalline lens and avoid tissue ablation [5,6].

Numerous studies have established both the IPCL and the ICL as effective, well-tolerated options. However, lens selection should be individualized based on refractive error, age, ocular parameters, and patient expectations [7,8].

In the present study, postoperative outcomes following implantation of the IPCL V2.0 and ICL V4c in eyes with more myopia than -5.00 D were evaluated. Fifty-five eyes were enrolled (27 IPCLs, 28 ICLs), and baseline demographics were comparable (mean age: 23.70 versus 24.78 years; p = 0.5823). The current findings align with those of Rateb et al., Yu et al., and Sachdev et al., all of whom reported similar age distribution across lens types and no baseline differences [9-11].

The current findings show a female predominance in both groups, consistent with the gender trends noted in earlier reports [9,10,12]. The male-to-female ratio was 1:2 in the IPCL group and 1:1 in the ICL group. This pattern reflects a higher uptake of refractive surgery among women, as noted in several studies.

Visual outcomes in both groups showed significant improvement. In Group A (IPCL), LogMAR-UCVA improved from 1.411 ± 0.121 preoperatively to a 12-month postoperative UCVA of 0.268 ± 0.202. In Group B (ICL), UCVA improved from 1.366 ± 0.217 preoperative to a 12-month postoperative UCVA of 0.146 ± 0.133. Within-group comparisons revealed statistically significant gains in both cohorts (p < 0.00001). However, intergroup analysis demonstrated that Group B achieved better visual outcomes at 12 months (p = 0.0477), suggesting a potential advantage of ICL in terms of refractive precision. The superior visual outcomes observed with the ICL group may be related to factors such as superior optical quality, more accurate refractive correction, better lens design and sizing, better long-term stability, and fewer optical aberrations. However, these mechanisms were not directly evaluated in the present study, and therefore this interpretation should be considered speculative.

These findings are consistent with those of Rateb et al. and Yu et al., who reported excellent visual outcomes with both lenses [9,10]. However, the current results showed a more favourable UCVA in the ICL group, suggesting a potential edge in visual quality -although longer follow-up is needed to assess long-term stability.

Sachdev et al. reported median postoperative LogMAR-BCVA of 0.0 (IQR = 0.0-0.1) for both IPCL and ICL groups at one year (p = 0.038) [11]. Their study noted line gain in 31.1% of IPCL-treated eyes and 26.1% of ICL-treated eyes. Although both lenses demonstrated similar efficacy, the IPCL was considered an effective and economically viable option for myopia correction. These findings align with the present study's outcomes.

Taneri et al. observed that, at three months postoperatively, 83% of IPCL-treated eyes, and 92% of ICL-treated eyes achieved UCVA equal to or better than preoperative BCVA [12]. The visual efficacy of both lenses was comparable, consistent with the present findings.

In the current analysis, mean ACD across both groups was 2.903 ± 0.390 mm, and mean vault at one month postoperatively was 598.89 ± 129.38 μm. A strong, positive correlation was observed between ACD and vaulting (R2 = 0.5643; p < 0.0001). This suggests that greater ACD values are predictive of higher postoperative vaults. Several studies support this association, also confirming ACD as a predictor of vault height [13-17].

In contrast, WTW measurements in the current study (mean: 11.574 ± 0.447 mm) did not significantly correlate with vault height (R2 = 0.0106; p = 0.4548). This suggests that WTW, measured via slit lamp with a Verneir calliper, may not be a reliable predictor of vaulting. Similar results were noted by Yang et al. and Trancón et al., who concluded that angle-to-angle (ATA) diameter is a more robust predictor of vault compared to WTW [13,14]. Studies by Igarashi et al. and Zheng et al. further supported the limited utility of WTW in isolation, advocating for multi-parameter biometric models [15,18]. In the present study, anterior chamber depth showed a significant correlation with postoperative vault, whereas WTW distance did not. This finding suggests that ACD may better reflect the internal anterior segment anatomy and the space available for P-IOL implantation, thereby exerting a greater influence on the achieved vault. In contrast, WTW is an external corneal measurement and may not accurately represent the true ciliary sulcus diameter owing to inter-individual anatomical variability. This limitation may explain the absence of a significant association between WTW and postoperative vault in our cohort. These findings highlight the limitations of WTW-based sizing alone and suggest that internal ocular anatomical parameters may be more predictive of postoperative vault. Consequently, reliance on WTW alone may reduce the accuracy of lens sizing. Additionally, the present study did not include ATA, anterior chamber angle (ACA), or lens curvature, which limits the ability to model vault prediction comprehensively.

Zheng et al. reported a mean vault of 0.33 ± 0.19 mm three months postoperatively, measured by ultrasound biomicroscopy (UBM) [15]. Vaulting correlated significantly with ACA, WTW, and lens curvature (Lenscur). However, WTW alone demonstrated weak predictive value (R2 = 0.061; p = 0.029). A multivariable regression model incorporating multiple anterior segment parameters was required to predict vaulting effectively. In contrast, the current study did not include ACA or Lenscur, limiting predictive modelling.

Alfonso et al. found a positive correlation between WTW and vault (R2 = 0.29; p < 0.001), while Seo et al. reported significantly higher vaults in high WTW eyes (0.57 ± 0.36 mm) compared to low WTW eyes (0.25 ± 0.14 mm; p = 0.01), with WTW showing stronger correlation than ACD or sulcus diameter [16,17]. These results differ from the present findings, in which no significant correlation between WTW and vault was observed. Appropriate sizing of posterior chamber P-IOL is essential for achieving optimal postoperative vault and minimizing complications. Although UBM-derived sulcus-to-sulcus (STS) measurement is generally regarded as the anatomical reference for P-IOL sizing because it directly measures the ciliary sulcus, its routine use is limited by equipment availability, operator dependence, additional examination time, and patient discomfort. Therefore, WTW and ACD-based sizing remains the most practical and widely adopted approach in routine clinical practice and was used in the present study in accordance with the manufacturer's sizing recommendations.

During the 12-month follow-up, 14.5% of eyes developed grade 1-2 anterior uveitis and IOP elevation at one week postoperatively. These cases responded to topical steroids, cycloplegics, and beta-blockers. Isolated case reports have described similar IOP elevations requiring long-term antiglaucoma therapy or surgical intervention [18-20]. Cycloplegics such as cyclopentolate may help reduce vault and prevent angle narrowing, as suggested by prior studies [21].

Halos and glare were reported in 25.4% of eyes (29.6% IPCL, 21.4% ICL) during late follow-up. These symptoms are well-documented following P-IOL implantation and may persist beyond six months [22]. Maroccos et al. found that smaller optic diameters increased the risk of photic disturbances [23]. Other studies demonstrated symptom reduction with brimonidine tartrate 0.2% and identified risk factors, including pupil-optic zone mismatch and ICL toricity [24,25]. Several recent studies support these findings, highlighting the comparable visual efficacy of both IPCL and ICL models [9-12,26,27].

Both reinforced acrylic and Collamer posterior chamber P-IOL provided significant visual improvement over a 12-month period in patients with myopia of more than ˗5.00D. While both designs were effective and well-tolerated, the Collamer lens with four haptic pads demonstrated superior uncorrected visual acuity outcomes. Despite this advantage, the reinforced acrylic lens remained a potentially lower-cost alternative, though affordability was not assessed, offering meaningful refractive correction. The present findings have important clinical implications for posterior chamber P-IOL implantation. Although WTW- and ACD-based sizing remains the most widely adopted clinical approach, multimodal sizing strategies incorporating anterior segment OCT and UBM-derived STS measurements may further improve vault predictability in selected patients. Long-term follow-up remains essential to monitor endothelial cell density, cataract formation, intraocular pressure, and vault stability. Future prospective studies with larger sample sizes, longer follow-up, and incorporation of advanced anterior segment imaging modalities are warranted to optimize lens sizing algorithms and further evaluate the long-term safety and effectiveness of posterior chamber P-IOL implantation.

Study limitations

This study was conducted at a single tertiary centre with a limited sample size, restricting generalizability. Only two P-IOL designs were evaluated, and long-term follow-up is required to assess the stability. Due to limited resources available, there was an absence of important parameters, such as ATA/ACA/lens curvature, endothelial cell density/endothelial cell loss, assessment of cataract formation, pigment dispersion, and lack of corneal safety outcome. There was an absence of subgroup analysis of glare/halo complication between toric and non-toric implants due to the small sample size and lack of cost comparison and affordability assessment between IPCL Group A and ICL Group B.

## Conclusions

This study demonstrated significantly promising visual outcomes postoperatively over a period of one year in both the ICL model Visian V4c and the IPCL model V2.0 for the surgical correction of myopia. Though the result of the ICL model Visian V4c was found to be significantly better than the IPCL model V2.0, IPCL remained a potentially lower-cost material as compared to the ICL model, taking into account that affordability was not assessed. This study concluded that there was a significantly strong positive correlation between ACD and postoperative vaulting, whereas an insignificant correlation between WTW and postoperative vaulting. Both the IPCL and ICL are effective and safe options for correcting high myopia with comparable complication rates.
